# Physical activity and polluted air: implications for COPD prevention and policy

**DOI:** 10.1016/j.lanwpc.2025.101693

**Published:** 2025-09-18

**Authors:** Omar Hahad, Karsten Keller, Jos Lelieveld

**Affiliations:** a Department of Cardiology – Cardiology I, University Medical Center of the Johannes Gutenberg–University Mainz, Mainz, Germany; b German Center for Cardiovascular Research (DZHK), Partner Site Rhine-Main, Mainz, Germany; c Max Planck Institute for Chemistry, Mainz, Germany; d Climate and Atmosphere Research Center, The Cyprus Institute, Nicosia, Cyprus

Regular physical activity reduces the risk of chronic obstructive pulmonary disease (COPD)^1^ and improves the prognosis in affected patients.^2^ In contrast, ambient air pollution, is a substantial risk factor for COPD worldwide.^3^ On a global scale, COPD attributable to air pollution was responsible for an estimated 1.78 (95% CI 1.42–2.17) million deaths and 37.52 (30.52–44.89) million disability-adjusted life years (DALYs) in 2021.^4^ This substantial health burden raises questions about how air pollution may interact with other health-related behaviors.

In *The Lancet Regional Health–Western Pacific*, Xia and colleagues provide robust evidence that high long-term exposure to fine particulate matter (PM_2.5_), nitrogen dioxide (NO_2_), and ozone (O_3_) can attenuate or even reverse the protective association of physical activity with COPD risk.^5^ The analysis, based on nearly half a million Chinese adults, demonstrated clear differences depending on local air pollution levels. In areas with relatively low exposure, higher total physical activity was associated with substantially reduced COPD risk, whereas in high-exposure areas these benefits diminished and, at the highest pollution levels, even shifted towards an excess risk. The modifying effect was particularly evident in women and in non-smokers.

These results are consistent with the biological plausibility that increased ventilation during physical activity increases deposition of inhaled pollutants in the lungs, thereby counteracting health benefits in heavily polluted environments.^6^^,^^7^ However, beyond mechanistic explanations, the central message of this study is its practical relevance for public health, clinical guidance, and policy development. In regions with chronic high air pollution levels, health promotion strategies must consider the balance between encouraging physical activity and reducing air pollutant exposure. Practical approaches may include^8^:

• promoting exercise during times of lower air pollution (for example early morning),
• shifting activity to green corridors and urban parks,
• improving access to affordable and well-ventilated indoor exercise facilities,
• and protecting outdoor workers through adapted schedules and occupational safeguards.

These findings highlight that improving air quality and promoting physical activity must be implemented together to achieve maximum health benefits. Current global recommendations, such as the World Health Organization (WHO) physical activity guidelines, do not explicitly address environmental modifiers such as air pollution.^9^ Emphasizing the role of air pollution would strengthen their applicability, particularly in low- and middle-income countries where exposures remain high.

National governments can take several steps, including^10^:

• Align clean air action plans with strategies to increase population-level activity.
• Incorporate environmental context into guidelines for COPD prevention and management.
• Expand urban planning measures that reduce traffic-related pollution and simultaneously create safe spaces for active living.

China's recent clean air initiatives show that substantial reductions in population exposure are achievable.^11^ Such efforts, if coupled with policies that facilitate active lifestyles, could reduce the COPD burden more effectively than either intervention alone. Similar approaches are needed in other rapidly urbanizing regions of Asia, Africa, and Latin America, where high pollution and low activity coexist.

Importantly, the interaction between air pollution and physical activity is not limited to COPD. Emerging evidence suggests similar patterns for cardiovascular outcomes and mortality.^7^ Integrating environmental exposures into lifestyle recommendations is therefore an important next step in preventive medicine. From a global health perspective, this means that policies to address non-communicable diseases must not be developed in isolation from environmental policies. Tackling both simultaneously will be crucial to reduce disease burden in the 21st century.

In summary, the study by Xia and colleagues provides timely and compelling evidence that the benefits of physical activity for COPD prevention are conditional on environmental context. In high-pollution settings, the protective association weakens and may even reverse. Clinicians, public health practitioners, and policymakers must therefore integrate environmental exposures into recommendations and planning. Only by combining efforts to improve air quality and promote physical activity can the global burden of COPD be effectively reduced.

## Contributors

OH, KK, and JL conceptualized the manuscript and wrote the first draft. OH, KK, and JL contributed to the interpretation of the literature and critically revised the manuscript. All authors approved the final version.

## Declaration of interests

None.
